# Gonorrhœa Speedily Relieved

**Published:** 1888-01

**Authors:** J. J. Buster

**Affiliations:** Mt. Willing, S. C.


### GONORRHŒA SPEEDILY RELIEVED.

BY J. J. BUSTER, M. D., MT. WILLING, S. C.

A few months since I was consulted by a young man in great distress. His first exclamation was "My God, Doctor, I'm ruined; I've got the clap and am to be married in ten (10) days." I advised him, if possible, to have his marriage postponed for at least two weeks, which he said was not possible.

On examination I found the usual symptoms very prominent with an unusual œdema and excessive discharge.

Ordered for him a basin of hot water, and told him to bathe for at least fifteen minutes; which he did, experiencing much relief.

I then made several injections of hot water into the urethra. He then urinated very freely, and with a marked diminution in pain. I then used Fl. Canadensis, (P. D. & Co.'s) ʒij, aqua qs. ℥i; injected into canal, and had him to hold it in for about three minutes. And in a half or three-quarters of an hour, I used the following: subnit. ʒii bismuth ʒiv, aqua ℥i; thrown into the urethra by elevating the piston end of the syringe, and with very gentle force passing it in. Kept solution in, for I suppose, five minutes. Ung. belladonna from Prostatic portion to glands externally. Gave potassa bicarb, gr. xx, every four to six hours, with advice to return on the following day.

In due time he put in his appearance, looking much more cheerful. To my great surprise the discharge had stopped. Redness and swelling had almost entirely disappeared, and with no pain at all in urinating. I applied the ung. belladonna again as before, and renewed his supply of potassa bicarb. His marriage was consummated at the stated time, and no trouble has ever arisen since, (of a gonorrhœal character).

Have used the same treatment in several cases since, and in no case has it been troublesome to relieve.

